# LDL suppresses angiogenesis through disruption of the HIF pathway via NF-κB inhibition which is reversed by the proteasome inhibitor BSc2118

**DOI:** 10.18632/oncotarget.4943

**Published:** 2015-09-15

**Authors:** Gang Yao, Qi Zhang, Thorsten R. Doeppner, Feng Niu, Qiaochuan Li, Yanping Yang, Ulrike Kuckelkorn, Nina Hagemann, Wei Li, Dirk M. Hermann, Yun Dai, Wen Zhou, Fengyan Jin

**Affiliations:** ^1^ Cancer Center, the First Affiliated Hospital, Jilin University, Changchun, Jilin, China; ^2^ Department of Neurology, the Second Affiliated Hospital, Jilin University, Changchun, Jilin, China; ^3^ Department of Neurology, University Hospital Essen, Essen, Germany; ^4^ Department of Hematology, the First Affiliated Hospital, Guangxi Medical University, Nanning, Guangxi, China; ^5^ Department of Biochemistry, Charité Universitätsmedizin Berlin, Berlin, Germany; ^6^ Department of Medicine, Virginia Commonwealth University, Massey Cancer Center, Richmond, Virginia, USA; ^7^ Cancer Research Institute, Central South University, Key Laboratory of Carcinogenesis and Cancer Invasion, Ministry of Education, Key Laboratory of Carcinogenesis, National Health and Family Planning Commission, Changsha, Hunan, China

**Keywords:** low-density lipoprotein, HIF, NF-κ B, proteasome inhibitor, angiogenesis

## Abstract

Since disturbance of angiogenesis predisposes to ischemic injuries, attempts to promote angiogenesis have been made to improve clinical outcomes of patients with many ischemic disorders. While hypoxia inducible factors (HIFs) stimulate vascular remodeling and angiogenesis, hyperlipidemia impairs angiogenesis in response to various pro-angiogenic factors. However, it remains uncertain how HIFs regulate angiogenesis under hyperlipidemia. Here, we report that exposure to low-density lipoprotein (LDL) suppressed *in vitro* angiogenesis of human brain microvascular endothelial cells. Whereas LDL exposure diminished expression of HIF-1α and HIF-2α induced by hypoxia, it inhibited DMOG- and TNFα-induced HIF-1α and HIF-2α expression in normoxia. Notably, in both hypoxia and normoxia, LDL markedly reduced expression of HIF-1β, a constitutively stable HIF subunit, an event associated with NF-κB inactivation. Moreover, knockdown of HIF-1β down-regulated HIF-1α and HIF-2α expression, in association with increased HIF-1α hydroxylation and 20S proteasome activity after LDL exposure. Significantly, the proteasome inhibitor BSc2118 prevented angiogenesis attenuation by LDL through restoring expression of HIFs. Together, these findings argue that HIF-1β might act as a novel cross-link between the HIF and NF-κB pathways in suppression of angiogenesis by LDL, while proteasome inhibitors might promote angiogenesis by reactivating this signaling cascade under hyperlipidemia.

## INTRODUCTION

Hypoperfusion decreases blood flow through an organ, which if prolonged, may result in permanent cellular dysfunction and death. To this end, numerous attempts have been made to stimulate vascular remodeling under the conditions of hypoperfusion, in order to improve outcomes of patients with ischemic disorders. Among them, the approaches for ischemic preconditioning (IPC) or stabilizing HIF-α (e.g., by inhibition of HIF-specific prolyl hydroxylases, PHDs) offer potential therapeutic strategies to treat various ischemic disorders, including peripheral artery occlusive disease, myocardial infarction, and stroke. [1–6] HIF-1α, a protein expressed ubiquitously in virtually all cell types, governs cellular responses to hypoxia, including initiation of angiogenesis. [7–9] HIF-2α is selectively expressed in certain cell types, particularly in vascular endothelial cells, which therefore has recently implicated in regulation of angiogenesis and neovascularization. [10, 11] In hypoxia, HIF-α subunits form the heterodimers with the constitutively stable subunit HIF-1β (also known as ARNT) to induce transcription of genes with diverse adaptive functions. However, under well-oxygenated conditions, HIF-α subunits are hydroxylated at the conserved proline residues, which are then recognized by the von Hippel-Lindau protein (pVHL) complex, an E3 ubiquitin ligase, leading to degradation via the 26S proteasome. [7–9, 12] Moreover, NF-κB increases HIF-1β stabilization, [13, 14] suggesting a potential role of NF-κB in regulation of the HIF pathways in ischemic diseases. [6, 15]

However, vascular remodeling remains a challenge particularly in patients with severe atherosclerosis. [16–18] Atherosclerosis is known to associate with hypercholesterolemia that causes deposition of lipids into the blood vessel wall. For example, about half of patients with ischemic stroke have hypercholesterolemia, [19] while human subjects with hypercholesterolemia often have high risk of stroke mortality. [20, 21] In this context, increased cholesterol levels can trigger a number of vascular abnormalities, such as oxidative stress, incapacitated vasodilation, inflammation, as well as impaired angiogenesis. [22–24]

To address the clinical issue that ischemic diseases occur frequently in atherosclerosis patients with hyperlipidemia, we hypothesized that blood lipids might impair angiogenic responses to hypoxia, TNFα, or PHD inhibitors, via inhibition of the HIF pathways. The present studies provide evidence for the role of LDL in attenuating angiogenic response of endothelial cells to hypoxia, DMOG (dimethyloxaloylglycine, an inhibitor of PHD) or TNFα, in association with down-regulation of HIFs, most likely through promoting their proteasomal degradation. Of note, it is the first time, to the best of our knowledge, to show that the proteasomal inhibitor BSc2118 restore LDL-attenuated angiogenesis by reactivation of HIF-1α and HIF-2α.

## RESULTS

### Native, but not oxidized, LDL attenuates angiogenesis of endothelial cells, an event associated with down-regulation of HIF-1α, HIF-2α, and HIF-1β

**Figure 1:**
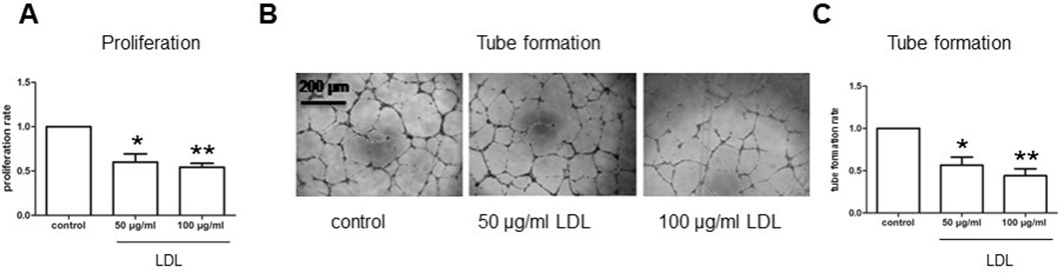
LDL attenuates cell proliferation and tube formation of hCMEC/D3 cells under hypoxic condition The immortalised human brain microvascular endothelial cell line hCMEC/D3 cells cultured in Microvascular Endothelial Cell Medium-2, containing either 0.5% FBS for proliferation assay or 5% FBS for tube formation assay, were treated (72 hr) with the indicated concentrations of native LDL under hypoxic (1% oxygen) condition, after which cells were subjected to the following assays: **A.** cell proliferation assay using the BrdU Flow Kit; **B–C.** Matrigel-based tube formation assay. Three independent experiments (*n* = 3) were performed. *p < 0.05, **p < 0.01 versus controls. Scale bars, 200 μm.

**Figure 2:**
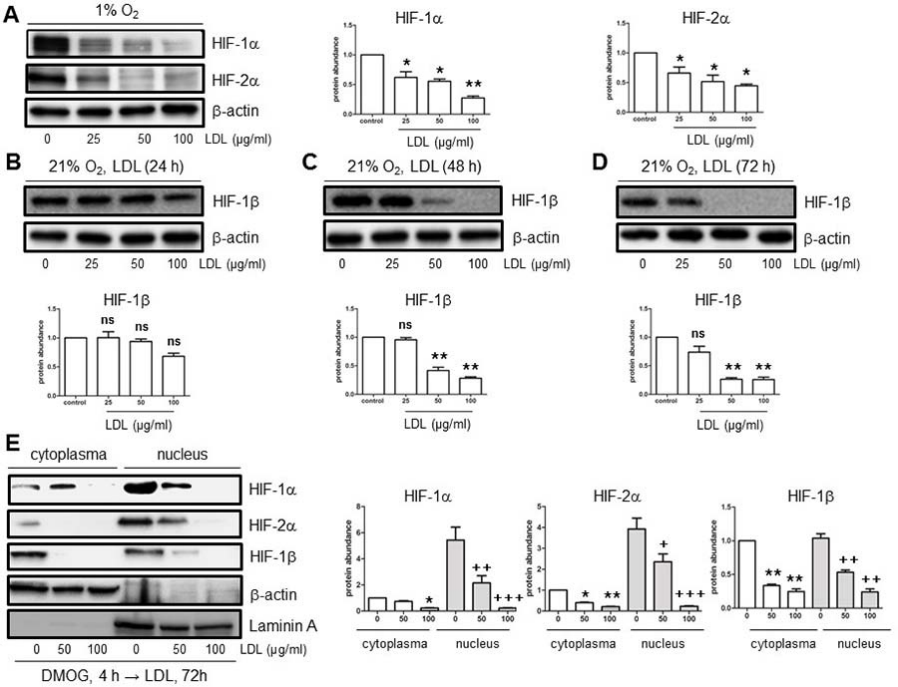
LDL down-regulates HIF-1α, HIF-2α, and HIF-1β in hCMEC/D3 cells in both hypoxia and normoxia **A.** hCMEC/D3 cells were exposed to the indicated concentrations of native LDL (25–100 μg/ml) under hypoxic (1% O_2_) condition for 72 hr, after which Western blot analysis was performed to monitor protein levels of HIF-1α and HIF-2α. **B–D.** hCMEC/D3 cells were exposed to the indicated concentrations of LDL for 24 (B), 48 (C), and 72 hr (D) under normoxic (21% O_2_) condition, after which Western blot analysis was performed to monitor protein levels of HIF-1β. **E.** hCMEC/D3 cells were treated with 1 μM DMOG for 4 hr, followed by 50–100 μg/ml LDL for additional 72 hr, after which expression of HIF-1α, HIF-2α, and HIF-1β in the cytoplasmic and nuclear fractions was assessed by Western blot analysis. Blots re-probed for β-actin and laminin A were used as loading controls for cytoplasmic and nuclear fractions, respectively. All blots were quantified densitometrically using ImageJ software. The relative protein abundance was calculated by comparing to either β-actin or Laminin A and expressed as fold increase over controls (without LDL treatment). Values for controls were arbitrarily set to 1.0. At least three independent experiments (*n* ≥ 3) were performed. *p < 0.05, **p < 0.01, ***p < 0.001 (for whole cell lysates or cytoplasmic fraction); ^+^*p* < 0.05, ^++^*p* < 0.01, ^+++^*p* < 0.001 (for nuclear fraction) versus their controls; ns, not significant.

To test our hypothesis that the HIF family is involved in attenuation of angiogenesis under hyperlipidemia, we first examined whether LDL impairs angiogenesis induced by hypoxia using an immortalised human brain microvascular endothelial cell line (hCMEC/D3) as a model. hCMEC/D3 cells were exposed to 50–100 μg/ml native LDL under hypoxic (1% O_2_) condition, after which cell proliferation and Matrigel-based tube formation assays were performed. As shown in Fig. 1, native form of LDL markedly attenuated both proliferation (Fig. 1A) and tube formation (Fig. 1B–1C) of hCMEC/D3 cells in hypoxia. Western blot analysis revealed that whereas hypoxia markedly induced expression of HIF-1α and HIF-2α (Suppl. Fig. 1), native LDL significantly reduced the protein abundance of HIF-1α and HIF-2α in hypoxia (Fig. 2A). Notably, while HIF-1β was constitutively expressed regardless of oxygen availability (i.e., in both normoxia and hypoxia; Suppl. Fig. 1), native LDL reduced expression of HIF-1β under normoxic condition (Fig. 2B–2D). In contrast, oxidized LDL (ox-LDL) failed to reduce mRNA levels of HIF-1β (Suppl. Fig. 2A). Moreover, native LDL also attenuated protein levels of HIF-1α, HIF-2α, HIF-1β in cytoplasmic and especially nuclear fraction of hCMEC/D3 cells treated with the prolyl hydroxylase (PHD) inhibitor dimethyloxalylglycine (DMOG, an agent known to induce HIF activation; [26] Fig. 2E), suggesting that LDL might prevent dimerization of HIF-1β with HIF-1α or HIF-2α and thus inhibit their transcriptional activation in the nuclei. However, ox-LDL was not able to alter protein levels of HIF-1α, HIF-2α, and HIF-1β in hCMEC/D3 cells treated with DMOG (Suppl. Fig. 2B). Further, the free radical scavenger phenyl-N-tert-butyLnitrone (PBN) [27] failed to prevent down-regulation of HIF-1α and HIF-2α by LDL in hCMEC/D3 cells treated with DMOG (Suppl. Fig. 2C), supporting the notion that oxidation might be not required for LDL to inhibit HIF activation. However, whereas no effect on mRNA levels of HIF-1α and HIF-2α was noted (Suppl. Fig. 3A–3B), LDL increased, rather than reduced, HIF-1β mRNA expression in hCMEC/D3 cells with or without DMOG (24 h in particular; Suppl. Fig. 3C–3D), probably reflecting a compensatory response to down-regulation of HIF-1β protein. Together, these findings argue that in normoxia, native LDL (but not oxidized LDL) inhibits angiogenesis likely through down-regulation of HIF-1β. They also raise a possibility that the mechanism(s) other than inhibition of *de novo* gene expression might be involved in HIF-1β down-regulation by LDL.

### LDL prevents TNFα-induced expression of HIFs through inactivation of the NF-κB pathway in endothelial cells

**Figure 3:**
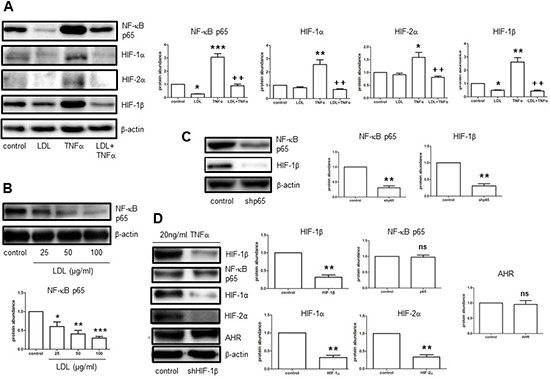
LDL inhibits NF-κB-dependent expression of HIF-1β induced by TNFα in hCMEC/D3 cells, resulting in HIF-1α and HIF-2α down-regulation in normoxia **A.** hCMEC/D3 cells were exposed to either LDL (100 μg/ml) alone or in combination with TNFα (20 ng/ml), after which Western blot analysis was performed to monitor expression of NF-κB p65, HIF-1α, HIF-2α, and HIF-1β. **B.** hCMEC/D3 cells were incubated with the indicated concentrations of LDL for 72 hours, after which protein levels of NF-κB p65 in whole cell lysates was determined by Western blot analysis. **C.** hCMEC/D3 cells were transiently transduced with pLKO. 1 *NF-κB p65* shRNA (shp65), followed by Western blot analysis for detecting protein levels of NF-κB p65 and HIF-1β. **D.** hCMEC/D3 cells were transiently transduced with pLKO.1 *HIF-1β* shRNA (shHIF-1β), and then exposed to TNFα, after which Western blot analysis was performed to monitor protein levels of HIF-1β, NF-κB p65, HIF-1α, HIF-2α, and AHR. At least three independent experiments (*n* ≥ 3) performed. *p < 0.05, **p < 0.01, ***p < 0.001 versus controls without LDL treatment; ^+^*p* < 0.05, ^++^*p* < 0.01 versus controls with TNFα treatment alone; ns, not significant.

Ischemia induces production of TNFα, which in turn promotes cerebral angiogenesis. [15] In this context, we observed that TNFα sharply induced expression of HIF-1β, while moderately increased protein levels of HIF-1α and HIF-2α in hCMEC/D3 cells (Fig. 3A). These events were accompanied by activation of the NF-κB pathway, reflected by a marked increase in protein expression of p65, a key component of the most abundant NF-κB p65/p50 heterodimer, in hCMEC/D3 cells exposed to TNFα, a classical NF-κB agonist. Moreover, co-administration of either the NF-κB inhibitor PDTC or the IKK inhibitor Bay 11-7082 largely prevented TNFα-induced expression of HIF-1α, HIF-2α, and HIF-1β at protein level in hCMEC/D3 cells (Suppl. Fig. 4A–4D). Similarly, LDL diminished both NF-κB activation (e.g., inhibition of p65 induction) and up-regulation of HIF-1α, HIF-2α, and particularly HIF-1β in response of hCMEC/D3 cells to TNFα (Fig. 3A). Of note, LDL also decreased basal levels of NF-κB p65 in a dose-dependent manner in hCMEC/D3 cells (Fig. 3B). To validate the role of the NF-κB pathway in this setting, NF-κB p65 was knocked down using shRNA. Indeed, down-regulation of p65 by shRNA mimicked the capability of LDL to inhibit HIF-1β expression in hCMEC/D3 cells (Fig. 3C). In contrast, shRNA knockdown of HIF-1β failed to decrease NF-κB p65 expression (Fig. 3D). These findings argue that LDL down-regulates expression of HIF-1β via a NF-κB-dependent process.

### HIF-1β acts as a cross-link between inhibition of NF-κB and attenuation of HIF-1α/HIF-2α expression in endothelial cells exposed to LDL

Up-regulation of HIF-1α expression by NF-κB represents an important mechanism that governs the HIF pathway. [13, 14] While inhibitors of the NF-κB pathway (e.g., PDTC, Bay 11-7082) blocked TNFα-induced expression of HIF-1α, HIF-2α, and HIF-1β (Suppl. Fig. 4A–4D), qPCR revealed that NF-κB inhibition (e.g., by Bay 11-7082) only abrogated up-regulation of HIF-1β mRNA in hCMEC/D3 cells exposed TNFα (Suppl. Fig. 5A–5C). Interestingly, whereas TNFα did not induce mRNA expression of either HIF-1α or HIF-2α, Bay 11-7082 failed to reduce their mRNA levels in hCMEC/D3 cells with or without induction by TNFα. Thus, these results raise a possibility that HIF-1α, as well as HIF-2α, might not be up-regulated directly by NF-κB. To this end, it was observed that HIF-1β knockdown by shRNA decreased protein expression of both HIF-1α and HIF-2α in the presence of TNFalpha in hCMEC/D3 cells, while did not alter NF-κB p65 abundance (Fig. 3D). However, the protein level of aryl hydrocarbon receptor (AHR), known as a binding partner of HIF-1β, was not changed after HIF-1β knockdown (Fig. 3D). These findings support a notion that LDL suppresses HIF-1β gene expression through inhibition of the NF-κB pathway, which in turn specifically results in reduction of HIF-1α and HIF-2α protein levels. Thus, HIF-1β may act as a cross-link between the NF-κB and HIF signaling pathways, as well as play a crucial role in inhibition of the HIF family by LDL.

### LDL induces HIF-1α proline hydroxylation and promotes 20S proteasome activity in endothelial cells in both normoxia and hypoxia

**Figure 4:**
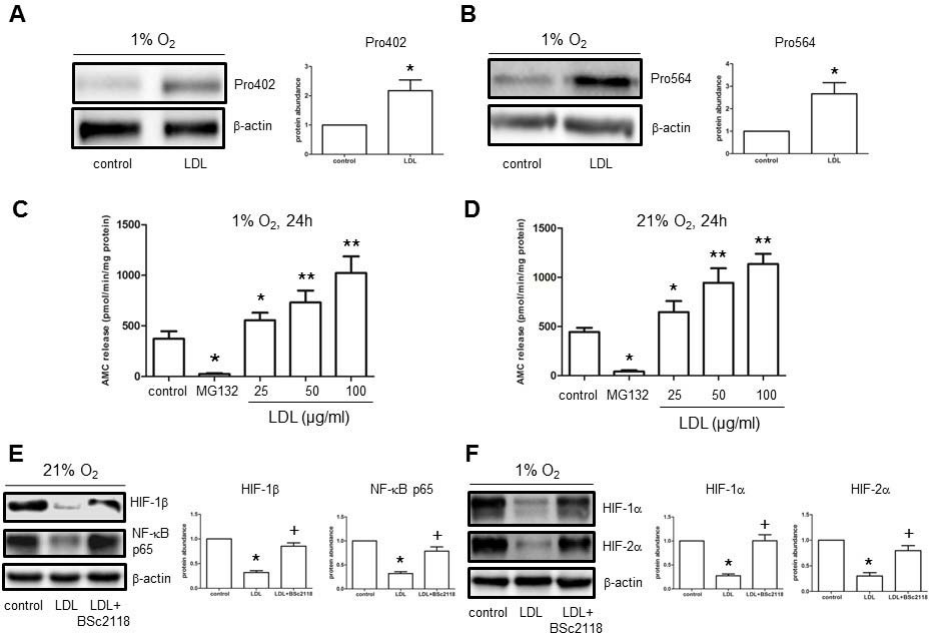
LDL induces HIF-1α hydroxylation at Pro402 and Pro564 sties, while increases 20S proteasome activity in hCMEC/D3 cells **A–B.** hCMEC/D3 cells were exposed to LDL (100 μg/ml) in hypoxia, after which Western blot analysis was performed to monitor hydroxylation of HIF-1α using antibodies specifically recognizing hydroxylated HIF-1α at Pro402 (A) and Pro564 (B) respectively. **C–D.** CT-L activity of 20S proteasome was analysed in hCMEC/D3 after exposed (24 hr) to the indicated concentrations of LDL in either hypoxia (C) or normoxia (D). Cells were treated with MG132 as control. **E–F.** hCMEC/D3 cells were exposed to 100 μg/ml LDL for 48 hr in the absence or presence of pre-treatment with 100 nM BSc2118 (4 hr prior to LDL) in normoxia (E) or hypoxia (F), after which Western blot analysis was performed to assess expression of NF-κB p65 and HIF-1β. At least three independent experiments (*n* ≥ 3) were performed. *p < 0.05, **p < 0.01 versus controls without LDL treatment; ^+^*p* < 0.05 versus controls with LDL treatment alone.

In well-oxygenated environments, HIF-1α proline hydroxylation is critical for recognition by E3 ubiquitin ligase in the pVHL complex, followed by degradation via the ubiquitin-proteasome system (UPS). [28] In this context, LDL reduced abundance of HIF-1α protein (Fig. 2A, 2E, and 3A), but not mRNA (Suppl. Fig. 5A), raising a possibility that HIF-1α down-regulation by LDL might involve its turnover through the UPS. Thus, the effect of LDL on HIF-1α proline hydroxylation was examined. Under hypoxic condition, exposure to LDL resulted in a marked increase in proline hydroxylation of HIF-1α at both Pro402 and Pro564 residues in hCMEC/D3 cells (Fig. 4A–4B), accompanied by sharply increased chymotrypsin-like activity of 20S proteasome in a dose-dependent manner (Fig. 4C). Similar phenomena were also observed under normoxic condition (Fig. 4D). Significantly, administration of the proteasome inhibitor BSc2118 restored abundance of both NF-κB p65 and HIF-1β in cells exposed to LDL in normoxia (Fig. 4E). Moreover, BSc2118 also reversed attenuation of HIF-1α and HIF-2α expression by LDL under hypoxic condition (Fig. 4F). Together, these findings argue that LDL induces HIF-1α degradation via the UPS, in association with increased proline hydroxylation as well as 20S proteasome activity. They also suggest that inhibition of the UPS by proteasome inhibitors (e.g., BSc2118) might reverse impairment of angiogenesis by LDL via restoration of HIF (HIF-1α, HIF-2α, HIF-1β) expression.

### The proteasome inhibitor BSc2118 restores LDL-attenuated angiogenesis of endothelial cells, in association with reactivation of the HIF pathway

**Figure 5:**
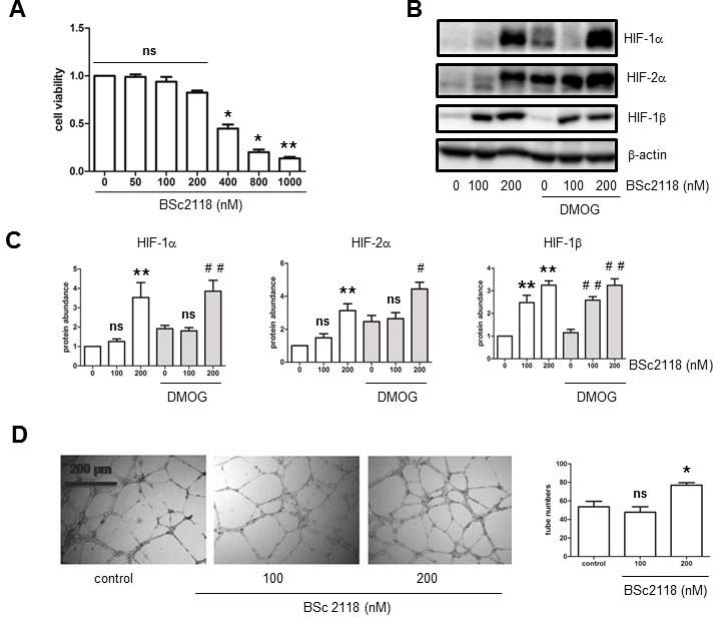
The proteasome inhibitor BSc2118 induces tube formation of hCMEC/D3 cells, in association with HIF-1α and HIF-2α accumulation **A.** hCMEC/D3 cells were exposed to the indicated concentrations of BSc2118 for 24 hr, followed by cell viability assay. **B–C.** hCMEC/D3 cells were exposed to BSc2118 (100–200 μg/ml) with or without DMOG for 4 hr, after which Western blot analysis was performed to monitor protein levels of for HIF-1α, HIF-2α, and HIF-1β. **D.** hCMEC/D3 cells were treated with 100–200 nM BSc2118, followed by Matrigel-based tube formation assay. Scale bars, 200 μm. Three independent experiments (*n* = 3) were performed. *p < 0.05, **p < 0.01 versus control without BSc2118; ^#^*p* < 0.05, ^##^*p* < 0.01 versus controls with DMOG alone; ns, not significant.

**Figure 6:**
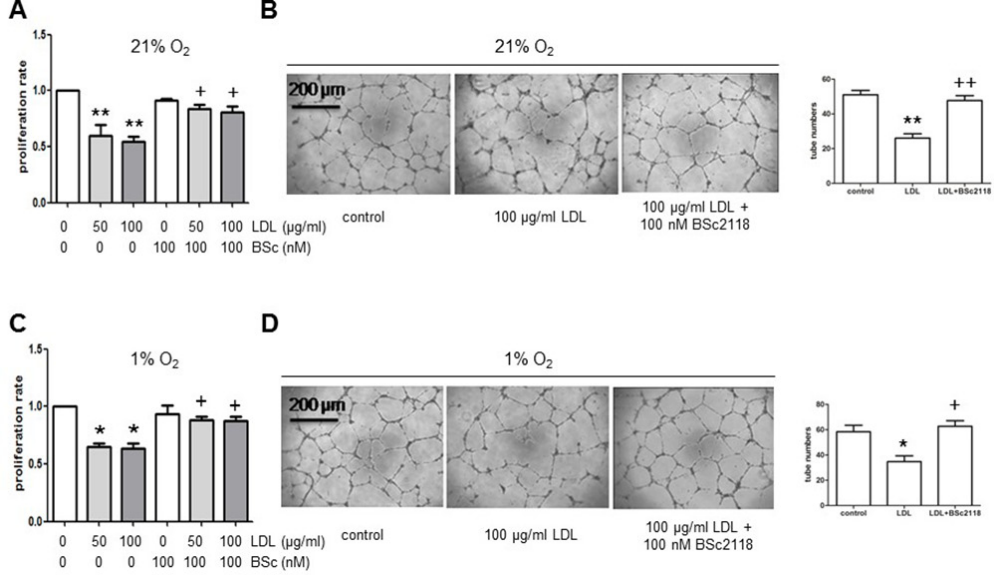
The proteasome inhibitor BSc2118 restores LDL-attenuated proliferation and tube formation of hCMEC/D3 cells in both normoxia and hypoxia **A–B.** hCMEC/D3 cells were exposed to 50–100 μg/ml of LDL in the absence or presence of 100 nM BSc2118 in normoxia, after which cell proliferation analysis (A) and Matrigel-based tube formation assay were carried out. **C–D.** Alternatively, hCMEC/D3 cells were treated as described in panel A-B in hypoxia, and then subjected to analyses of cell proliferation and tube formation. Three independent experiments (*n* = 3) were performed. *p < 0.05, **p < 0.05 versus controls without LDL treatment; ^+^*p* < 0.05 versus controls with LDL treatment alone. Scale bars, 200 μm.

Finally, to examine whether up-regulation of HIFs by proteasome inhibitors could reverse attenuation of angiogenesis in hyperlipidemia, the effects of BSc2118 on hCMEC/D3 cells after exposed to LDL were analyzed. First, the dose-response experiments were performed to determine the non-toxic doses of BSc2118 towards hCMEC/D3 cells. As shown in Fig. 5A, although BSc2118 concentrations ≥ 400 nM were cytotoxic, the doses ≤ 200 nM of this agent had virtually no effect on viability of hCMEC/D3 cells. The latter nontoxic doses were then chosen to further conduct the following experiments. Second, Western blot analysis revealed that administration of 200 nM BSc2118 resulted in marked increases in proteins levels of HIF-1α, HIF-2α, and HIF-1β in hCMEC/D3 cells, with or without induction by DMOG (Fig. 5B–5C). Notably, lower concentrations (e.g., 100 nM) of BSc2118 also strikingly induced expression of HIF-1β, but not HIF-1α and HIF-2α (Fig. 5B–5C). These results further support the critical role of HIF-1β in regulation of the HIF pathway. HIF-1α and HIF-2α accumulation also reflected a synergistic effect between DMOG and BSc2118 (Fig. 5B–5C). Third, consistent with up-regulation of HIFs, the non-toxic concentrations (e.g., 200 nM) of BSc2118 significantly promoted tube formation of hCMEC/D3 cells (Fig. 5D). Last, although there was a little effect by itself (Fig. 5D), lower concentrations (e.g., 100 nM) of BSc2118 also restored LDL-attenuated angiogeneisis of hCMEC/D3 cells reflected by increased cell proliferation and tube formation, in both normoxia (Fig. 6A–6B) and hypoxia (Fig. 6C–6D). Together, these findings indicate that the proteasome inhibitor BSc2118 is able to reverse inhibition of angiogenesis by LDL, most likely through reactivation of the HIF pathway. They also raise a possibility that proteasome inhibitors (e.g., BSc2118) might be effective to treat patients with ischemic diseases, particularly those related to hyperlipidemia such as atherosclerosis.

## DISCUSSION

Ischemic diseases often correlate to hyperlipidemia (e.g., in atherosclerosis patients), due to deficiency of angiogenesis in response to hypoxia in the latter circumstances. [29] Herein, we hypothesized that blood lipids such as LDL might inhibit angiogenic responses. Indeed, the present study demonstrated that exposure to native LDL, rather than its oxidized form, markedly suppressed proliferation and tube formation of endothelial cells in an *in vitro* model of angiogenesis using hCMEC/D3 cells, an immortalised human brain microvascular endothelial cell line. Moreover, it was found that attenuation of endothelial angiogenesis by LDL was associated with disruption of the HIF pathway, including down-regulation of HIF-1α and HIF-2α, in hypoxia. Furthermore, it was also identified that HIF-1β plays a central role in regulation of the HIF pathway in this setting. In this case, LDL down-regulated HIF-1β at transcriptional level through inhibition of NF-κB, leading to inactivation of the HIF pathway (e.g., down-regulation of HIF-1α and HIF-2α) and the resulting attenuation of angiogenesis in responses to various pro-angiogenic factors, including hypoxia, DMOG, and TNFα. Thus, these findings might provide a new insight into the mechanisms underlying defective angiogenesis in hyperlipidemia.

In the present study, up-regulation (activation) of HIF-1α and HIF-2α was not observed in response to the PHD inhibitor DMOG in endothelial cells after exposed to LDL. However, expression of HIF-1β, a constitutively stable subunit that is ubiquitously expressed and forms a heterodimer with HIF-α, was dramatically decreased after exposure to LDL, even in the presence of DMOG. Further, nuclear localization of HIF-1α, HIF-2α, and HIF-1β was diminished by LDL in cells treated with DMOG, suggesting perturbations of HIF-α/HIF-1β heterodimerization and transcriptional activation in the nuclei, an event required for induction of angiogenesis. On the other hand, hypercholesterolemia has been shown to impair angiogenesis *in vivo* by inducing oxidative stress in blood vessels. [22] Moreover, oxidized LDL has been found to inhibit endothelial cell proliferation and migration *in vitro*, in association with decreased NO synthase and Akt activity in endothelial cells. [30–32] However, it is noteworthy that the native form of LDL displayed anti-angiogenic activity, consistent with those reported earlier. [33, 34] Surprisingly, oxidized LDL failed to reduce abundance of HIF-1α, HIF-2α, and HIF-1β, while the antioxidant PBN was not able to rescue LDL-induced down-regulation of HIFs. These results argue against the role of LDL oxidation in regulation of the HIF pathway in angiogenesis.

Emerging evidence indicates the cross-talk between the HIF and NF-κB pathways. For example, inflammatory stimuli like TNFα activate HIF-1α in a NF-κB-dependent manner. [13, 14] Moreover, the role of NF-κB in HIF-1α regulation have implicated in ischemic diseases. [6] In the present study, it was found that TNFα not only induced HIF-1α expression, but also up-regulated HIF-2α and HIF-1β. However, disruption of NF-κB activation either by directly suppressing this pathway (e.g., by pharmacological inhibitors) or by LDL abrogated TNFα-induced up-regulation of HIF-1α, HIF-2α, and HIF-1β, while only blocked transcription of HIF-1β. Furthermore, whereas shRNA knockdown of NF-κB p65 directly down-regulated HIF-1β, genetic knockdown of HIF-1β diminished expression of both HIF-1α and HIF-2α. These findings further highlight a functional role of HIF-1β as a novel cross-link between the NF-κB and HIF signaling pathways.

As down-regulation of HIF-1α and HIF-2α did not occur at transcriptional level, a possibility arises that other mechanism(s) may be involved. To this end, it was observed that exposure to LDL clearly increased proline hydroxylation of HIF-1α, an event driving its recognition by E3 ligase in the VHL complex and subsequently degradation via the UPS. [7] Importantly, LDL also markedly promoted chymotrypsin-like activity of 20S proteasome in both hypoxia and normoxia. To the best of our knowledge, the phenomena that LDL induces down-regulation of HIFs through increasing HIF hydroxylation and proteasome activity have never been reported before, which might lay a basis for a therapeutic strategy to promote angiogenesis in hyperlipidemia by interference with these events.

Recently, it has been shown that proteasome inhibition has a neuroprotective effect in animal models such as those with middle cerebral artery occlusion. [35–37] Among others, the potential mechanisms involve inhibition of the NF-κB and toll-like receptor signaling pathways. [36–38] Significantly, the proteasome inhibitor BSc2118 prevented down-regulation of NF-κB p65 as well as HIF-1α, HIF-2α, and HIF-1β in endothelial cells exposed to LDL. Consistent with these novel findings, BSc2118 also restores LDL-attenuated proliferation and tube formation of endothelial cells. In this context, our previous study has demonstrated that treatment with BSc2118 leads to long-term neuroprotection. [38] Together, the present findings argue that the proteasome inhibitors (e.g., BSc2118) might promote angiogenesis, particularly in hyperlipidemia, via restoring activity of the HIF pathway. However, it cannot be ruled out that other proteins, particularly those subjected to degradation via the UPS, also contribute to the mechanism of action of BSc2118 in this setting.

In summary, the present study using an *in vitro* angiogenesis model showed that whereas LDL attenuated angiogenesis in response to hypoxia through inactivation of the NF-κB-and HIF-1β-dependent HIF signaling pathway, the proteasome inhibitor BSc2118 was able to prevent LDL-mediated anti-angiogenesis by restoration of HIF activity. The former might provide a rationale to target anti-angiogenic effects of LDL that often occurs in patients with hyperlipidemia. Importantly, the latter findings lay a foundation for a novel therapeutic strategy using proteasome inhibitors (e.g., BSc2118) to treat ischemic diseases in patients with hyperlipidemia. This strategy may have particular relevance to conditions of chronic hypoperfusion derived from advanced atherosclerosis in organs like heart and brain. However, the present findings are solely based on an *in vitro* model of angiogenesis using a human endothelial cell line, which might not entirely reflect the physiological conditions in intact animals, while acute exposure to LDL might differ from chronic hyperlipidemia status in patients. Thus, whereas the succeeding *in vivo* studies in living animals is required for further validation of these *in vitro* findings, proteasome inhibitors warrant consideration in the treatment of patients with ischemic diseases such as stroke or coronary heart disease, particularly those with hyperlipidemia.

## MATERIALS AND METHODS

### Cell culture

hCMEC/D3 cells, an immortalised human brain microvascular endothelial cell line, were kindly provided by Dr. Pierre-Olivier Couraud (Institut Cochin, INSERM, Paris), and propagated from passages 25 to 35 in Microvascular Endothelial Cell Medium-2 with 5% FBS (Lonza, Allendale, NJ, U.S.A.). Cells were cultured in a humidified incubator at 37°C and 5% CO_2_. 1% O_2_ condition used for all hypoxic experiments was achieved in a chamber with continuous infusion of pre-tested gas mixture containing 95% N_2_ and 5% CO_2_.

### Reagents

BSc2118, a proteasome inhibitor, was a gift from Dr. Kuckelkorn (Charité Universitätsmedizin). LDL and recombinant human tumor necrosis factor-α (TNFα) were purchased from Invitrogen (Carlsbad, CA) and Peprotech (Rocky Jill, NJ), respectively. The free radical scavenger phenyl-N-tert-butyLnitrone (PBN), the NF-κB inhibitor pyrrolidine dithiocarbamic acid (PDTC), the IκB kinase (IKK) inhibitor Bay 11-7082, the prolyl hydroxylase inhibitor dimethyloxalylglycine (DMOG), and the proteasome inhibitor MG132, were obtained from Sigma-Aldrich (St. Louis, MO). The antibodies used in this study include rabbit anti-human laminin A polyclonal antibody (Sigma-Aldrich); mouse anti-Human HIF-1α monoclonal antibody (BD Transduction Laboratories, San Jose, CA); human/rat HIF-2α antibody (R&D Systems, Minneapolis, MN); rabbit anti-human HIF-1β/ARNT polyclonal antibody, rabbit anti-human NF-κB p65 monoclonal antibody, rabbit anti-human β-actin monoclonal antibody (Cell Signaling Technology, Danver, MA); and anti-HIF-1α (hydroxylated Pro402 and Pro564) antibody (EMD Millipore, Billerca, MA). The NE-PER Nuclear Protein Extraction Reagent Kit was purchased from Pierce (Life Technologies, Frederick, MD).

### Plasmids and lentivirus transduction

*E.coli* XL1-blue (Stratagene, Heidelberg, Germany) was generally used for plasmid preparation, while *E.coli* Stbl3 (Invitrogen) were used for preparation of pLKO.1 (8453, Addgene, Cambridge, MA), pLKO.1-shRNA-HIF-1α, pLKO.1-shRNA-HIF-2α, pLKO.1-shRNA-HIF-1β, and pLKO.1-shRNA-p65 (Sigma-Aldrich). Recombinant lentivirus was produced as described previously. [25] hCMEC/D3 cells were infected with lentivirus and then selected. Western blot analysis was performed to monitor gene silencing.

### LDL oxidation

Briefly, native LDL was dialysed against 1× PBS for 24 hours and then incubated with 10 μM CuSO_4_ for 24 hours at 37°C, followed by dialysis against 1× PBS containing 0.1 mM EDTA for 48 hours. Oxidized LDL was harvested through a 0.22 μm filter and used within one week.

### Cell viability and proliferation assays

2 × 10^4^ hCMEC/D3 cells suspended in Microvascular Endothelial Cell Medium-2 containing 0.5% serum were seeded into 24-well plates. LDL (50–100 μg/ml) and BSc2118 (50–1000 nM) were added either alone or in combination. For the latter, BSc2118 was added 4 hours prior to LDL. After incubation for 24 hours, cell viability was analyzed using the Cell Counting Kit-8 (CCK-8; Dojindo Laboratories, Kumamoto, Japan). Alternatively, after incubation for 72 hours, cell proliferation was monitored using the BrdU Flow Kit (BD Biosciences, Franklin Lakes, NJ).

### Endothelial cell tube formation assay

2 × 10^4^ hCMEC/D3 cells suspended in Microvascular Endothelial Cell Medium-2 containing 5% serum were seeded into 96-well plates pre-coated with Matrigel (BD Biosciences, Franklin Lakes, NJ). Cells were then incubated in normoxia (21% O_2_) or hypoxia (1% O_2_), in the absence or presence of 50–100 μg/ml LDL +/− 100 nM BSc2118 (pre-treatment for 4 hours) as described above. After 24 hours, images (magnification, 5 ×) were captured in a 5% CO_2_ incubator using a microscope with a DFC-290 camera (Leica Microsystems, Wetzlar, Germany).

### *In vitro* proteasome activity assay

Chymotrypsin-like (CT-L) proteasome activity assay was conducted using the 20S Proteasome Assay Kit, SDS-Activated (EMD Millipore) as per the manufacturer's instructions.

### Western blot analysis

After exposed to LDL, TNFα, BSc2118, or other pharmacological inhibitors, or transduced with lentivirus vectors as described above for the indicated intervals, hCMEC/D3 cells were harvested. Whole cell lysates were prepared in NP40 lysis buffer containing protease inhibitor cocktail (Roche, Deutschland, Germany). Whole cell lysates or cellular fractions (prepared as above) were resolved on 7.5% sodium dodecyl sulfate-polyacrylamide gels (SDS-PAGE) and blotted onto polyvinylidene fluoride (PVDF) membranes. Membranes were then blocked with 5% skimmed milk in TBS-T (tris-buffered saline containing 0.5% Tween 20, pH 7.2), followed by incubation with the primary antibodies (see “Reagents”). Blots were visualized with the Enhanced Chemiluminescence (ECL) Kit (Pierce, Life Technologies). Blots were re-probed with anti-β-actin (Cell Signaling Technology) to ensure equal protein loading.

### Qualitative real-time-PCR (qPCR)

hCMEC/D3 cells were pretreated with PDTC or Bay 11-7082 for 24 hours, followed by exposed to LDL, DMOG, TNFα alone or in combination with LDL for the indicated intervals. qPCR analysis using the ABI PRISM 7500 Real Time PCR System (Applied Biosystems, Foster City, CA) were performed to quantify mRNA levels of human target genes. Briefly, total RNA was extracted using the RNeasy Midi Kit (Qiagen, Valencia, CA) as per the manufacturer's instructions. cDNA was synthesized from 1 μg of total RNA. Gene expression was then analyzed by two-step real-time PCR: 95°C for 30 seconds, followed by 40 cycles of 95°C for 5 seconds, and 60°C for 34 seconds. The following primers were used: *Hif-1α*, 5′-CAAGGGCCACCAGAAGCGCAA-3′, 3′-TTTGGAGAGGATCTTGAGGCTGGA-5′; *Hif-2α*, 5′-AGGACTACAGCCTGTCGTCAGC-3′, 3′-CCTTGCA GGAGCGTGGAG-5′; *Hif-1β*, 5′-GTGGCAGTAGCT CTGTGGACC-3′, 3′-AGCCAAGTCCATTCCTGCAT-3′; *NF-κB p65*, 5′-CCAGACCAACAACAACCCCT-3′, 3′-GATCTTGAGCTCGGCAGTGT-3; *Gapdh*, 5′-CATCT TCCAGGAGCGAGATCC-3′, 3′-GGTGCAGGTGGCA TTGCTGATG-5′. Reference for quantitation was the human housekeeping gene *Gapdh*. All PCR reactions were run in triplicate, and gene expression relative to *Gapdh* was calculated using the 2^−ΔΔCT^ method.

### Statistical analysis

Values represent the means ± SD for at least three independent experiments performed in triplicate. Significance of differences between experimental variables was determined using the Student's *t* test (two-sided) or One-way ANOVA with Tukey Post-Hoc Test. *P* < 0.05 was considered statistically significant. All statistical analyses utilized the GraphPad Prism 5.

## SUPPLEMENTARY MATERIAL FIGURES


