# Supplementary material for: LDL suppresses angiogenesis through disruption of the HIF pathway via NF-κB inhibition which is reversed by the proteasome inhibitor BSc2118
Source: Oncotarget. 2015 Sep 15;6(30):30251–62. doi: 10.18632/oncotarget.4943 (PMC4745795; doi:10.18632/oncotarget.4943)
Supplement: Supplementary file 1 [file oncotarget-06-30251-s001.pdf]

SUPPLEMENTARY FIGURES

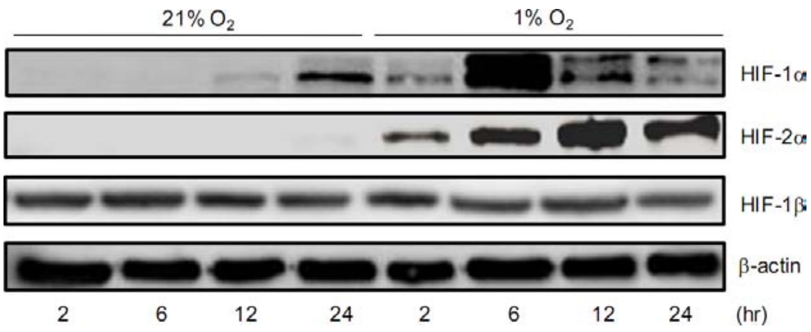

Supplementary Figure S1: Hypoxia induces expression of HIF-1α, HIF-2α, and HIF-1β in endothelial cells. hCMEC/D3 cells were cultured under either normoxic (21% O_2) or hypoxic (1% O_2) condition for the indicated intervals, after which Western blot analysis was performed to monitor protein levels of HIF-1α,HIF-2α and HIF-1β.

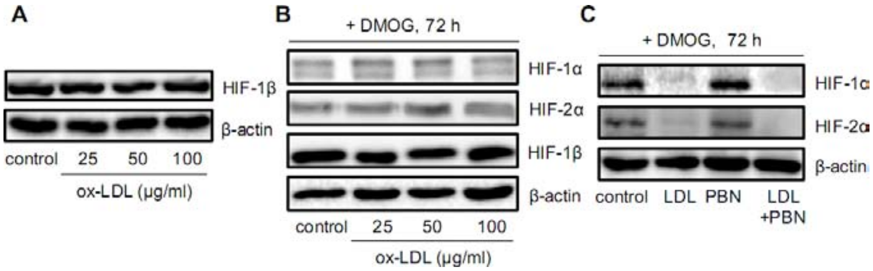

Supplementary Figure S2: Oxidised LDL (ox-LDL) fails to down-regulate HIF in endothelial cells in the presence or absence of DMOG. **A–B.** hCMEC/D3 cells were exposed to the indicated concentrations of ox-LDL (25–100 μg/ml) for 72 hr in the absence (A) or presence (B) of 1 μM DMOG, after which protein levels of HIF-1β, HIF-1α, and HIF-2α were assessed by Western blot analysis. **C.** hCMEC/D3 cells were pre-treated (4 hr) with the free radical scavenger phenyl-N-tert-butyInitrone (PBN), followed by exposure to 100 μg/ml LDL in the presence of 1 μM DMOG, after which Western blot analysis was performed to monitor expression of HIF-1α and HIF-2α. At least three independent experiments were performed.

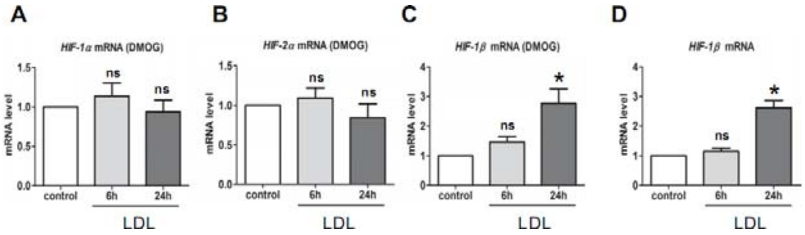

Supplementary Figure S3: LDL induces mRNA expression of HIF-1β, but not HIF-1α and HIF-2α, in hCMEC/D3 cells. A–B. hCMEC/D3 cells were exposed to100 µg/ml LDL for the indicated intervals (6 -24 hr) in the presence of DMOG, after which qPCR analysis was performed to monitor mRNA level of HIF-1α (A) and HIF-2α (B), respectively. **C–D.** hCMEC/D3 cells were treated with 100 µg/ml LDL for the indicated intervals (6 -24 hr) in the presence (C) or absence (D) of DMOG, after which HIF-1β mRNA levels were then assessed by qPCR. At least three independent experiments ($n \geq 3$) were performed. *$p < 0.05$ versus controls without LDL treatment; ns, not significant.

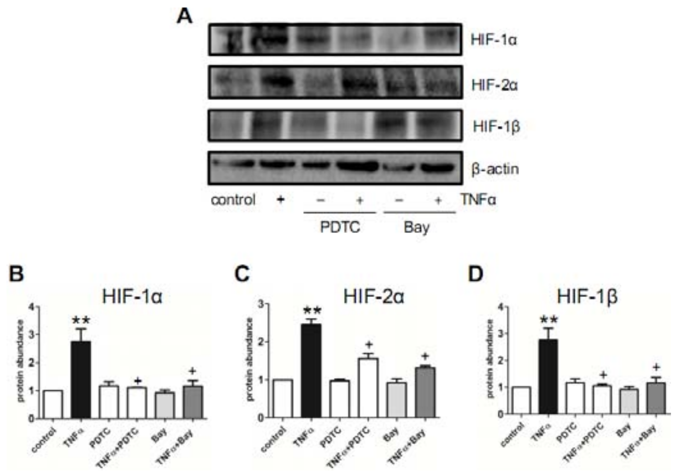

Supplementary Figure S4: Inhibition of the NF-κB signalling pathway blocks TNFα-induced HIF expression. A–D. hCMEC/D3 cells were pre-treated with the NF-κB inhibitor PDTC (50 μM) or the IKK inhibitor Bay 11-7082 (20 μM) for 24 hr, followed by 20 ng/ml TNFα for additional 4 hr, after which Western blot analysis was performed to monitor expression of HIF-1α, HIF-2α, and HIF-1β. Three independent experiments ($n = 3$) were performed. $^{**}p < 0.01$ versus controls without TNFα treatment; $^{+}p < 0.05$ versus TNFα alone.

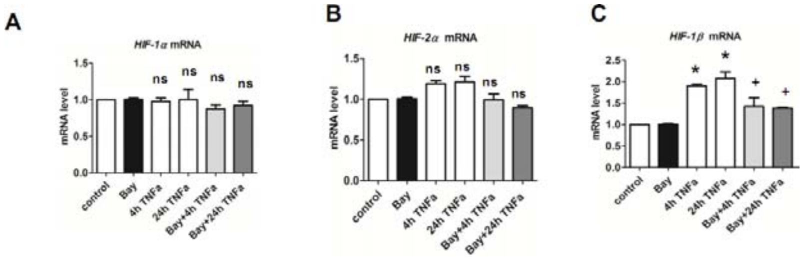

Supplementary Figure S5: TNFα induces only mRNA expression of HIF-1β, an event blocked by NF-κB inhibition, but not HIF-1α and HIF-2α. A–C. hCMEC/D3 cells were pre-treated with Bay 11-7082 (20 μM) for 24 hr, followed by 20 ng/ml TNFα for additional 4 or 24 hr, after which qPCR was carried out to determine mRNA levels of HIF-1α, HIF-2α, and HIF-1β, respectively. At least three independent experiments ($n \geq 3$) were performed. $^{*}p < 0.05$ versus controls without TNFα; $^{+}p < 0.05$ versus controls without Bay 11-7082; ns, not significant.
